# Synthesis and Cytotoxic Activity of Novel Tetrahydrocurcumin Derivatives Bearing Pyrazole Moiety

**DOI:** 10.1007/s13659-017-0143-9

**Published:** 2017-11-01

**Authors:** Ahmed Mahal, Ping Wu, Zi-Hua Jiang, Xiaoyi Wei

**Affiliations:** 1 Key Laboratory of Plant Resources Conservation and Sustainable Utilization/Guangdong Provincial Key Laboratory of Applied Botany, South China Botanical Garden, Chinese Academy of Sciences, Xingke Road 723, Tianhe District, Guangzhou, 510650 People’s Republic of China; 2 Department of Chemistry, Lakehead University, 955 Oliver Road, Thunder Bay, ON P7B 5E1 Canada

**Keywords:** Condensation, Tetrahydrocurcumin, Pyrazole, Anticancer agents, Drug discovery

## Abstract

**Abstract:**

Tetrahydrocurcumin (THC) is a major metabolite of curcumin and plays an important role in curcumin-induced biological effects. THC is a promising preventive and chemotherapeutic agent for cancer. A series of new pyrazole derivatives of THC have been synthesized as potent anticancer agents. Direct condensation of THC with various substituted hydrazines leads to new pyrazole derivatives of THC (**1**–**18**). The prepared compounds have been evaluated via in vitro MTT (3-(4,5-dimethylthiazol-2-yl)-2,5-diphenyltetrazolium bromide) assay for their cell proliferation-inhibitory activity against human lung adenocarcinoma (A549), human cervical carcinoma (HeLa) and human breast carcinoma (MCF-7) cells. Most derivatives show significantly higher anticancer activity against all three tested cancer cell lines than the parent compound THC. Several compounds (**7**, **8**, **12**, **13** and **15**) display promising anticancer activity against MCF-7 cell line with IC_50_ values ranging from 5.8 to 9.3 µM. The most active compound (**8**) is substituted with 4-bromophenyl group at the pyrazole ring and inhibits the growth of all three tested cancer cell lines with an IC_50_ values of (8.0 µM, A549), (9.8 µM, HeLa) and (5.8 µM, MCF-7). The obtained compounds can be a good starting point for the development of new lead molecules in the fight against cancer.

**Graphical Abstract:**

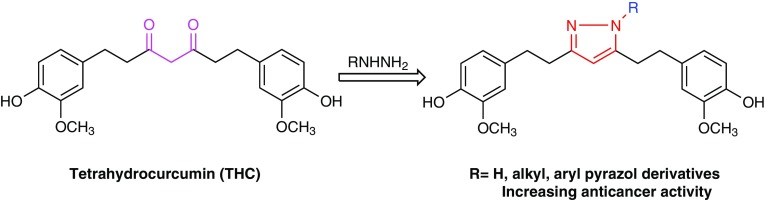

## Introduction

**Fig. 1:**
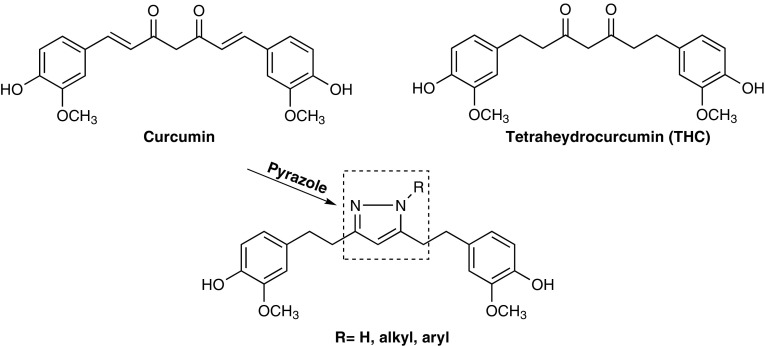
Structures of curcumin, tetrahydrocurcumin (THC) and pyrazole derivatives of THC

Cancer is one of the major causes of death worldwide despite remarkable progress in understanding the mechanism of the disease and finding appropriate treatments to control the disease and prevent death. A large number of natural products have been reported to show anticancer and cancer preventive activities. One good example is the naturally occurring yellow pigment curcumin (Fig. 1), which was isolated from the rhizomes of the plant *Curcuma longa* Linn, a member of the ginger family (Zingiberaceae) [1]. In vitro and in vivo research as well as clinical studies have shown the anticancer effect of curcumin as an anticancer and chemo-prevention agent [2, 3]. Curcumin has also been shown to act as a drug transporter-mediated MDR reversal agent [4, 5]. Tetrahydrocurcumin (THC, Fig. 1) is a major metabolite derived from curcumin. Reduction of curcumin (Fig. 1) by endogenous reductase system leads to THC which plays an important role in curcumin-induced biological effects [6]. THC can also be chemically synthesized from curcumin by catalytic hydrogenation using PtO_2_ or palladium as a catalyst [7, 8]. THC has been reported to inhibit tumor metastasis [9] and tumor angiogenesis in nude mice [10]. In 2007, Limtrakul and his coworkers demonstrated that THC acted as a MDR modulator when combined with other chemotherapeutics [11]. Furthermore, THC has protective effect on renal damage resulted from chemotherapy such as cisplatin for the treatment of cancer [12]. In addition to anticancer activity, THC displayed potent antioxidant activity [13], antidiabetic, anti-inflammatory, antiatherosclerotic and antihepatotoxic effects [14].

A large number of curcumin derivatives and analogues have been synthesized in order to improve its low bioavailability, pharmacological properties and therapeutic efficacy for the treatment of a number of diseases [15, 16]. In contrast, despite the great therapeutic potential THC has shown, so far few derivatives and analogues of THC have been synthesized in order to improve its biological activities [7, 17–21]. In fact, THC might be considered advantageous over curcumin as a starting material for structural modification in that the former is stable under physiological pH while the latter is not. A recent report suggests that curcumin is a highly improbable drug lead due to its instability, high reactivity and low bioavailability [22]. Here we choose THC as the staring material for preparing pyrazole derivatives.

In the past few decades, pyrazole and its derivatives have been attracted considerable attention due to the growing importance of their medical uses, particularly in Oncology. For example, some pyrazole derivatives have been shown to be highly potent against human breast [23], lung [24] and cervical [25] cancer cells. In the present study we have synthesized a group of new pyrazole derivatives of THC (Fig. 1) as potential anticancer agents. Replacing the 1,3-diketone by a pyrazole group may lead to increased anticancer activity.

## Results and Discussion

### Chemistry

**Scheme. 1:**
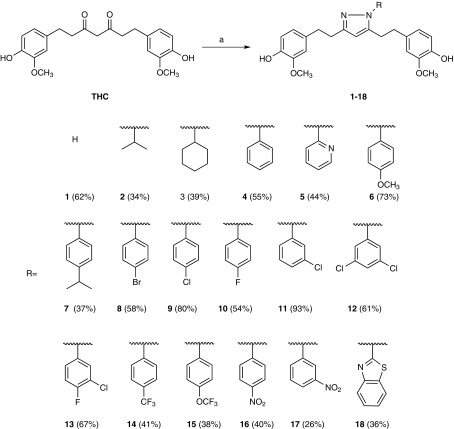
Synthesis of pyrazole derivatives of THC (**1**–**18**). Reagents and Conditions: *a* RNHNH_2_, HOAc, MeOH, reflux, 10 h

The synthesis of pyrazole derivatives of THC was carried out according to the previously reported method [26]. In general, the commercially available THC was subjected to the condensation reaction with substituted hydrazines involving the construction of two C–N bonds to afford substituted pyrazole derivatives (Scheme 1). The reaction was carried out by using catalytic amount of glacial acetic acid in absolute methanol under reflux for ten hours to afford the products. The yield of aryl-substituted pyrazole derivatives seems to be affected by the relative electron density of the aryl ring, which in turn is affected by the electron donating/withdrawing property of the substituent(s) on the aryl ring. Pyrazole derivatives with relatively more electron-rich aryl groups (**4**, **6**–**13**) were obtained in moderate to good yields while those with relatively more electron-poor aryl groups (**5**, **14**–**18**) in somewhat lower yields. Such trend can be readily understood because the decreased electron density on the nitrogen atoms of the substituted hydrazine would mean reduced nucleophilicity of the hydrazine and consequently lower yields of the products. The alkyl-substituted derivatives (**2** and **3**), however, were typically obtained in lower yields than their aryl-substituted counterparts, despite the electron-donating property of aliphatic groups. This phenomenon might be related to the fact that aryl-substituted pyrazoles are more stable than alkyl-substituted ones due to the resonance stabilization between the aryl ring(s) and the pyrazole ring. The condensation reaction of THC with unsubstituted hydrazine produced the known compound (**1**) [27] in 62% yield. All the prepared compounds were structurally confirmed by their ^1^H NMR, ^13^C NMR and MS data.

### Cytotoxic Activity

MTT (3-(4,5-dimethylthiazol-2-yl)-2,5-diphenyltetrazolium bromide) assay [28] was utilized in our study to evaluate in vitro cell proliferation-inhibitory activity of the prepared compounds against three cancer cell lines including human lung carcinoma (A549), human cervical carcinoma (HeLa) and human breast carcinoma (MCF-7). THC was used as the reference and the activity of all compounds is expressed as the concentration of drug at which 50% inhibition of cell growth (IC_50_) is achieved (Table 1). In general, most compounds show good anticancer activity against all three tested cancer cell lines. A few compounds appear to be more active against certain cell lines (e.g., **7**, **13** and **15** more active against MCF-7 cells, **16** more active against A549 cells, and **10** more active against HeLa cells).

**Table 1:** In vitro cell proliferation-inhibitory activity of the synthesized compounds (IC_50_, μM)^a^

Compound	Cell lines		
	A549	HeLa	MCF-7
**1**	> 50	36.4 ± 1.2	> 50
**2**	> 50	32.2 ± 0.5	> 50
**3**	14.1 ± 2.3	17.6 ± 1.5	12.1 ± 0.2
**4**	18.0 ± 2.3	19.2 ± 2.3	16.6 ± 0.6
**5**	> 50	> 50	28.7 ± 3.2
**6**	19.4 ± 0.3	20.1 ± 0.6	21.6 ± 2.2
**7**	15.1 ± 1.9	15.6 ± 1.3	**6.0** ± **0.7**
**8**	**8.0** ± **1.4**	**9.8** ± **0.8**	**5.8** ± **0.4**
**9**	14.1 ± 1.1	11.5 ± 1.5	11.1 ± 0.9
**10**	23.1 ± 0.3	10.2 ± 0.2	22.5 ± 2.7
**11**	12.6 ± 0.3	16.8 ± 1.7	15.5 ± 0.5
**12**	14.8 ± 1.1	13.0 ± 1.1	**9.3** ± **1.7**
**13**	18.8 ± 0.7	18.0 ± 0.5	**8.1** ± **1.2**
**14**	14.0 ± 1.9	**9.6** ± **1.8**	11.5 ± 0.5
**15**	13.8 ± 1.5	14.6 ± 1.6	**7.8** ± **0.6**
**16**	10.4 ± 0.7	15.2 ± 0.09	16.7 ± 3.2
**17**	13.3 ± 1.4	10.8 ± 1.8	15.9 ± 0.2
**18**	12.5 ± 1.5	29.9 ± 0.1	> 50
**THC**	> 50	33.6 ± 0.04	> 50

The derivatives of the significance [bold] showed significant activities against A-549, HeLa and MCF-7 cells

^a^ Values represent means ± SD based on three individual experiments

All aryl-substituted derivatives, except **5**, display significantly higher activity than the parent compound THC. The unsubstituted pyrazole derivative (**1**) and isopropyl-substituted pyrazole derivative (**2**) show no improvement in activity when compared to THC. Interestingly, the cyclohexyl-substituted derivative (**3**) shows much higher activity, which is comparable to the phenyl-substituted derivative (**4**), indicating that there is no real preference to either aliphatic or aromatic substitution on the pyrazole in terms of their cytotoxic activity. Surprisingly, the pyridinyl-substituted derivative (**5**) is much less active than the phenyl-substituted derivative (**4**), suggesting that the electronegative nitrogen atom in the pyridine ring might have played a negative role.

For these aryl-substituted derivatives, the substituent(s) on the aromatic ring can exert noticeable effect on their activity. For example, **15** (with 4-OCF_3_) is significantly more active than **6** (with 4-OCH_3_) against all cancer cell lines, indicating that the electron withdrawing effect of fluorine might have played a role. The effects of most other groups are not uniform across three cancer cell lines tested: for example, **16** (with 4-NO_2_) is more active than **10** (with 4-F) against A549 and MCF-7 cells but **10** is more active than **16** against HeLa cells. Among the 4-halogen-aryl-substituted derivatives (**8**–**10**), the 4-bromo-aryl compound (**8**) shows higher activity than 4-chloro-aryl (**9**) and 4-fluoro-aryl (**10**) compounds. The substitution position on the aryl ring does not seem to have much effect on the activity, e.g., the 4-chloro-aryl (**9**) vs. the 3-chloro-aryl (**11**) and the 4-nitro-aryl (**16**) vs. the 3-nitro-aryl (**17**). The 3,5-di-chloro-aryl compound (**12**) shows marginal effect on its activity in comparison with the 3-chloro-aryl compound (**11**), e.g., against MCF-7 cells, **12** has IC_50_ of 9.3 μM while **11** 15.5 μM. Similarly, the activity of 3-chloro-4-fluoro-aryl compound (**13**) is marginally shifted in one way or the other when compared to the corresponding mono-halogen substituted derivatives (**10** and **11**).

## Materials and Methods

### General Chemical Experimental Procedures

All reagents and solvents were purchased from commercial sources (Aladdin, Shanghai; Guangzhou Chemical Reagent Factory). Tetrahyrdocurcumin (THC) was purchased from Ark Pharm, Inc. Further purification and drying by standard methods were employed when necessary. Silica gel for column chromatography was purchased from Qingdao Marine Chemical Ltd., Qingdao, China. ^1^H and ^13^C NMR spectra were collected on a Bruker AVIII 500 M instrument at 500 (^1^H) and 125 (^13^C) MHz or a Bruker Avance-600 instrument at 600 (^1^H) and 150 (^13^C) MHz. Chemical shifts are reported as ppm (*δ* units) relative to tetramethylsilane (TMS) as an internal standard and the coupling constants as J in hertz (Hz). The splitting pattern abbreviations are as follows: s = singlet, d = doublet, dd = double doublet, t = triplet, m = multiplet, brs = broad singlet. ESIMS data were obtained on an MDS SCIEX API 2000 LC/MS instrument.

### General Procedure for the Synthesis of Compounds (**1**–**18**)

In a round bottom flask equipped with a magnetic bar and a condenser, tetrahydrocurcumin (1 eq.) in absolute methanol (1 mL) was added substituted hyrdrazines (2 eq.) and catalytic amount of glacial acetic acid. The reaction mixture was stirred under reflux for 10 h. The progress of reaction was monitored by TLC. The reaction was cooled to room temperature and concentrated in-vacuo and the residue poured in water. EtOAc (2 mL) was added to the mixture and aqueous layer was extracted three times with EtOAc (3 mL). The combined organic layers were washed with NaHCO_3_ and brine, dried over MgSO_4_ and concentrated in vacuo. The residue was purified by column chromatography (flash silica gel, DCM/EtOAc 99:1) to give the targets (**1**–**18**).

#### 3,5-Bis-[2-(4-hydroxy-3-methoxy)-phenylethyl]-1H-pyrazole (**1**)

White solid, yield: 62%. ^1^H NMR (500 MHz, CDCl_3_): *δ* = 6.71–6.70 (m, 4H, ArH), 6.60 (d, *J* = 1.72 Hz, 1H, ArH), 6.58 (d, *J* = 1.77 Hz, 1H, ArH), 5.83 (s, 1H, C4-H), 3.77 (s, 6H, OMe), 2.82 (bs, 8H, 4CH_2_); ^13^C NMR (400 MHz, CDCl_3_): *δ* = 149.3, 146.0, 137.6, 122.8, 117.1, 112.4, 103.3, 54.9, 35,1, 28.7; HR-ESI(-)MS m/z 367.1660 [M–H]^−^ (calcd for [C_21_H_23_N_2_O_4_]^−^, m/z 367.1663).

#### 1-(Isoprpyl)-3,5-bis-[2-(4-hydroxy-3-methoxy)-phenylethyl]-1H-pyrazole (**2**)

Colorless oil, yield: 34%. ^1^H NMR (500 MHz, CDCl_3_): *δ* = 6.71–6.70 (m, 4H, ArH), 6.60 (d, *J* = 1.72 Hz, 1H, ArH), 6.58 (d, *J* = 1.77 Hz, 1H, ArH), 5.83 (s, 1H, C4-H), 3.77 (s, 6H, OMe), 2.82 (bs, 8H, 4CH_2_); ^13^C NMR (400 MHz, CDCl_3_): *δ* = 149.3, 146.0, 137.6, 122.8, 117.1, 112.4, 103.3, 54.9, 35,1, 28.7; HR-ESI(-)MS m/z 409.2136 [M–H]^−^ (calcd for [C_24_H_29_N_2_O_4_]^−^, m/z 409.2132).

#### 1-(Cyclohexyl)-3,5-bis-[2-(4-hydroxy-3-methoxy)-phenylethyl]-1H-pyrazole (**3**)

Yellow oil, yield: 39%. ^1^H NMR (500 MHz, CDCl_3_): *δ *= 6.75 (d, *J* = 1.90 Hz, 1H, ArH), 6.71 (d, *J* = 1.34 Hz, 1H, ArH), 6.69 (d, *J* = 1.34 Hz, 1H, ArH), 6.66 (d, *J* = 1.34 Hz, 1H, ArH), 6.61 (dd, *J* = 4.13, 1.85 Hz, 1H, ArH), 6.42 (dd, *J* = 4.13, 1.89 Hz, 1H, ArH), 5.86 (s, 1H, C4-H), 3.81 (s, 3H, OMe), 3.78 (s, 3H, OMe), 3.76–3.73 (m, 1H, cyclohexyl), 2.88–2.81 (m, 8H, 4CH_2_), 1.32 (m, 7H, cyclohexyl), 1.34–1.28 (m, 3H, cyclohexyl); ^13^C NMR (400 MHz, CDCl_3_): *δ* = 151.2, 147.4, 147.3, 144.5, 144.1, 142.5, 133.3, 132.2, 120.6, 120.4, 114.7, 114.5, 111.9, 111.8, 102.6, 56.8, 55.0, 54.9, 35.5, 35.1, 32.5, 30.0, 27.1, 25.4, 25.0; HR-ESI(-)MS m/z 449.2437 [M–H]^−^ (calcd for [C_27_H_33_N_2_O_4_]^−^, m/z 449.2445).

#### 1-(Phenyl)-3,5-bis-[2-(4-hydroxy-3-methoxy)-phenylethyl]-1H-pyrazole (**4**)

Yellow oil, yield: 55%. ^1^H NMR (500 MHz, CDCl_3_): *δ* = 7.49–7.41 (m, 3H, ArH), 7.22–7.20 (m, 2H, ArH), 6.79 (d, *J* = 1.79 Hz, 1H, ArH), 6.72 (d, *J* = 7.78 Hz, 1H, ArH), 6.66–6.63 (m, 2H, ArH), 6.51 (d, *J* = 1.97 Hz, 1H, ArH), 6.51 (dd, *J* = 4.12, 1.89 Hz, 1H, ArH), 6.12 (s, 1H, C4-H), 3.82 (s, 3H, OMe), 3.72 (s, 3H, OMe), 2.89–2.86 (m, 6H, 3CH_2_), 2.89–2.72 (t, *J* = 7.16 Hz, 2H, CH_2_); ^13^C NMR (400 MHz, CDCl_3_): *δ* = 152.8, 147.4, 147.3, 144.5, 144.3, 144.2, 139.3, 133.0, 132.0, 128.7, 127.9, 125.7, 120.5, 120.4, 114.6, 111.8, 111.5, 104.6, 54.9, 54.8, 35.1, 34.6, 29.8, 27.8; HR-ESI (-)MS m/z 443.1973 [M–H]^−^ (calcd for [C_27_H_27_N_2_O_4_]^−^, m/z 443.1976).

#### 1-(Pyridine-2-yl)-3,5-bis-[2-(4-hydroxy-3-methoxy)-phenylethyl]-1H-pyrazole (**5**)

White solid, yield: 44%. ^1^H NMR (500 MHz, CDCl_3_): *δ* = 8.48–8.46 (m, 1H, ArH), 7.91–7.87 (m, 1H, ArH), 7.56–7.54 (m, 1H, ArH), 7.33–7.30 (m, 1H, ArH), 6.79 (d, *J* = 1.89 Hz, 1H, ArH), 6.72 (d, *J* = 8.02 Hz, 1H, ArH), 6.66–6.60 (m, 2H, ArH), 6.49 (dd, *J* = 4.14, 1.89 Hz, 1H, ArH), 6.10 (s, 1H, C4-H), 3.81 (s, 3H, OMe), 3.76 (s, 3H, OMe), 3.29 (t, *J* = 7.39 Hz, 2H, CH_2_), 2.92 (bs, 4H, 2CH_2_), 2.79 (t, *J* = 7.39 Hz, 2H, CH_2_); ^13^C NMR (400 MHz, CDCl_3_): *δ* = 153.9, 152.8, 147.5, 147.4, 147.3, 145.5, 144.4, 144.3, 138.5, 133.0, 132.5, 121.7, 120.5, 120.4, 117.1, 114.7, 114.6, 111.8, 111.6, 106.8, 54.9, 54.8, 34.9, 34.8, 29.9, 28.9; HR-ESI(-)MS m/z 444.1928 [M–H]^−^ (calcd for [C_26_H_26_N_3_O_4_]^−^, m/z 444.1928).

#### 1-(4-Methoxyphenyl)-3,5-bis-[2-(4-hydroxy-3-methoxy)-phenylethyl]-1H-pyrazole (**6**)

Red oil, yield: 73%. ^1^H NMR (500 MHz, CDCl_3_): *δ* = 7.07–7.05 (m, 2H, ArH), 6.97–6.95 (m, 2H, ArH), 6.78 (d, *J* = 1.79 Hz, 1H, ArH), 6.72 (d, *J* = 7.89 Hz, 1H, ArH), 6.65–6.63 (m, 2H, ArH), 6.48 (d, *J* = 1.80 Hz, 1H, ArH), 6.40 (dd, *J* = 4.13, 1.80 Hz, 1H, ArH), 6.60 (s, 1H, C4-H), 3.81 (s, 3H, OMe), 3.80 (s, 3H, OMe), 3.70 (s, 3H, OMe), 2.87 (bs, 4H, 2CH_2_), 2.79 (t, *J* = 7.11 Hz, 2H, CH_2_), 2.69 (t, *J* = 7.11 Hz, 2H, CH_2_); ^13^C NMR (400 MHz, CDCl_3_): *δ* = 159.6, 152.4, 147.4, 147.3, 144.5, 144.3, 133.1, 132.1, 132.0, 127.2, 120.6, 120.5, 114.7, 114.6, 113.8, 111.8, 111.5, 104.1, 54.9, 54.8, 54.6, 35.2, 34.6, 29.8, 27.7; HR-ESI(-)MS m/z 473.2085 [M–H]^−^ (calcd for [C_28_H_29_N_2_O_5_]^−^, m/z 473.2081).

#### 1-(4-Isopropylphenyl)-3,5-bis-[2-(4-hydroxy-3-methoxy)-phenylethyl]-1H-pyrazole (**7**)

Colorless oil, yield: 37%. ^1^H NMR (500 MHz, CDCl_3_): *δ* = 7.31 (d, *J* = 8.4 Hz, 2H, ArH), 7.11 (d, *J* = 8.4 Hz, 2H, ArH), 6.78 (d, *J* = 2.0 Hz, 1H, ArH), 6.72 (d, *J* = 7.89 Hz, 1H, ArH), 6.68–6.61 (m, 2H, ArH), 6.52 (d, *J* = 2.0 Hz, 1H, ArH), 6.41 (dd, *J* = 8.0, 2.0 Hz, 1H, ArH), 6.60 (s, 1H, C4-H), 3.81 (s, 3H, OMe), 3.71 (s, 3H, OMe), 2.87 (bs, 4H, 2CH_2_), 2.83 (t, *J* = 7.51 Hz, 2H, CH_2_), 2.70 (t, *J* = 7.51 Hz, 2H, CH_2_); ^13^C NMR (400 MHz, CDCl_3_): *δ* = 152.6, 149.0, 147.5, 147.4, 144.5, 144.4, 144.3, 137.0, 132.6, 126.7, 125.6, 120.5, 120.5, 114.7, 111.8, 111.6, 104.4, 54.9, 54.9, 35.2, 34.6, 33.6, 29.9, 27.8, 23.0; HR-ESI(-)MS m/z 487.2603 [M–H]^−^ (calcd for [C_30_H_35_N_2_O_4_]^−^, m/z 487.2591).

#### 1-(4-Bromophenyl)-3,5-bis-[2-(4-hydroxy-3-methoxy)-phenylethyl]-1H-pyrazole (**8**)

Red oil, yield: 58%. ^1^H NMR (500 MHz, CDCl_3_): *δ* = δ 7.58 (d, *J* = 8.7 Hz, 2H), 7.07 (d, *J* = 8.7 Hz, 2H), 6.79 (d, *J* = 1.9 Hz, 1H), 6.72 (d, *J* = 8.0 Hz, 1H), 6.67–6.60 (m, 2H), 6.48 (d, *J* = 1.9 Hz, 1H), 6.38 (dd, *J* = 8.0, 2.0 Hz, 1H), 6.13 (s, 1H), 3.82 (s, 3H), 3.71 (s, 3H), 2.88 (s, 6H), 2.72 (t, *J* = 7.3 Hz, 2H); ^13^C NMR (400 MHz, CDCl_3_): *δ* = 153.33, 147.4, 147.4, 144.60, 144.53, 144.33, 138.42, 133.02, 127.31, 121.32, 120.57, 120.53, 114.68, 114.67, 111.88, 111.58, 105.11, 55.00, 54.86, 35.07, 34.80, 29.80, 27.72; HR-ESI(-)MS m/z 521.1090 [M–H]^−^ (calcd for [C_27_H_26_BrN_2_O_4_]^−^, m/z 521.1081).

#### 1-(4-Chlorophenyl)- 3,5-bis-[2-(4-hydroxy-3-methoxy)-phenylethyl]-1H-pyrazole (**9**)

Orange oil, yield: 80%. ^1^H NMR (500 MHz, CDCl_3_): *δ* = 7.43–7.42 (m, 2H, ArH), 7.13–7.12 (m, 2H, ArH), 6.78 (d, *J* = 1.79 Hz, 1H, ArH), 6.72 (d, *J* = 7.86 Hz, 1H, ArH), 6.65–6.62 (m, 2H, ArH), 6.47 (d, *J* = 1.83 Hz, 1H, ArH), 6.40 (dd, *J* = 4.13, 1.81 Hz, 1H, ArH), 6.13 (s, 1H, C4-H), 3.81 (s, 3H, OMe), 3.70 (s, 3H, OMe), 2.87 (bs, 6H, 3CH_2_), 2.72 (t, *J* = 7.36 Hz, 2H, CH_2_); ^13^C NMR (400 MHz, CDCl_3_): *δ* = 153.2, 147.5, 147.4, 144.6, 144.5, 144.3, 137.9, 133.4, 133.0, 131.8, 128.8, 127.0, 120.6, 120.5, 114.7, 114.6, 111.8, 111.5, 105.0, 54.9, 54.8, 35.1, 34.7, 29.8, 28.8; HR-ESI(-)MS m/z 477.1580 [M–H]^−^ (calcd for [C_27_H_26_ClN_2_O_4_]^−^, m/z 477.1586).

#### 1-(3-Chlorophenyl)-3,5-bis-[2-(4-hydroxy-3-methoxy)-phenylethyl]-1H-pyrazole (**10**)

Yellow oil, yield: 54%. ^1^H NMR (500 MHz, CDCl_3_): *δ* = 7.40–7.39 (m, 2H, ArH), 7.15–7.11 (m, 2H, ArH), 6.79 (d, *J* = 1.87 Hz, 1H, ArH), 6.72 (d, *J* = 7.85 Hz, 1H, ArH), 6.65 (s, 1H, ArH), 6.63 (d, *J* = 7.85 Hz, 1H, ArH), 6.51 (d, *J* = 1.86 Hz, 1H, ArH), 6.39 (dd, *J* = 4.10, 1.81 Hz, 1H, ArH), 6.14 (s, 1H, C4-H), 3.81 (s, 3H, OMe), 3.71 (s, 3H, OMe), 2.89 (bs, 6H, 3CH_2_), 2.73 (t, *J* = 7.34 Hz, 2H, CH_2_); ^13^C NMR (400 MHz, CDCl_3_): *δ* = 153.4, 147.5, 147.4, 144.6, 144.5, 144.3, 140.3, 134.2, 133.0, 131.8, 130.0, 127.7, 125.5, 123.8, 120.6, 120.5, 114.7, 114.6, 111.8, 111.6, 105.2, 55.0, 54.8, 35.0, 34.7, 29.7, 27.7; HR-ESI(-)MS m/z 477.1589 [M–H]^−^ (calcd for [C_27_H_26_FN_2_O_4_]^−^, m/z 477.1586).

#### 1-(3,5-Dichlorophenyl)-3,5-bis-[2-(4-hydroxy-3-methoxy)-phenylethyl]-1H-pyrazol (**11**)

Red oil, yield: 93%. ^1^H NMR (500 MHz, CDCl_3_): *δ* = 7.42 (t, *J* = 1.87 Hz, 1H, ArH), 7.07 (d, *J* = 1.87 Hz, 2H, ArH), 6.79 (d, *J* = 1.83 Hz, 1H, ArH), 6.72 (d, *J* = 1.83 Hz, 1H, ArH), 6.64 (s, 1H, ArH), 6.63 (s, 1H, ArH), 6.50 (d, *J* = 1.81 Hz, 1H, ArH), 6.37 (dd, *J* = 4.10, 1.89 Hz, 1H, ArH), 6.17 (s, 1H, C4-H), 3.81 (s, 3H, OMe), 3.71 (s, 3H, OMe), 2.89 (bs, 6H, 3CH_2_), 2.73 (t, *J* = 7.11 Hz, 2H, CH_2_); ^13^C NMR (400 MHz, CDCl_3_): *δ* = 154.0, 147.5, 147.4, 144.8, 144.7, 144.3, 141.1, 134.9, 132.9, 131.5, 127.3, 123.8, 120.6, 120.5, 114.8, 114.7, 111.9, 111.5, 105.8, 55.0, 54.8, 35.0, 34.8, 29.7, 27.7; HR-ESI(-)MS m/z 511.1197 [M–H]^−^ (calcd for [C_27_H_26_ClN_2_O_4_]^−^, m/z 511.1196).

#### 1-(4-Flourophenyl)-3,5-bis-[2-(4-hydroxy-3-methoxy)-phenylethyl]-1H-pyrazole (**12**)

Orange oil, yield: 61%. ^1^H NMR (500 MHz, CDCl_3_): *δ* = 7.16 (d, *J* = 2.25 Hz, 2H, ArH), 7.15 (s, 2H, ArH), 6.79 (d, *J* = 1.80 Hz, 1H, ArH), 6.72 (d, *J* = 7.89 Hz, 1H, ArH), 6.65 (s, 1H, ArH), 6.63 (s, 1H, ArH), 6.49 (d, *J* = 1.80 Hz, 1H, ArH), 6.39 (dd, *J* = 4.37, 1.83 Hz, 1H, ArH), 6.11 (s, 1H, C4-H), 3.81 (s, 3H, OMe), 3.71 (s, 3H, OMe), 2.88 (bs, 4H, 2CH_2_), 2.83 (t, *J* = 7.29 Hz, 2H, CH_2_), 2.83 (t, *J* = 7.37 Hz, 2H, CH_2_); ^13^C NMR (400 MHz, CDCl_3_): *δ* = 162.0 (d, *J*_F,C_ = 247.3 Hz, CF), 152.9, 147.4, 144.6, 144.5, 144.3, 135.4 (d, *J*_F,C_ = 3.19 Hz, C), 133.0, 131.9, 127.8, 127.7, 126.9. 120.6, 120.5, 120.4, 115.5, 115.3, 114.6, 111.8, 111.5, 104.6, 54.9, 54.8, 35.1, 34.7, 29.8, 27.7; HR-ESI(-)MS m/z 461.1885 [M–H]^−^ (calcd for [C_27_H_25_Cl_2_N_2_O_4_]^−^, m/z 461.1882).

#### 1-(3-Chloro-4-flourophenyl)-3,5-bis-[2-(4-hydroxy-3-methoxy)-phenylethyl]-1H-pyrazole (**13**)

Red oil, yield: 67%. ^1^H NMR (500 MHz, CDCl_3_): *δ* = 7.29 (t, *J* = 8.74 Hz, 1H, ArH), 7.14 (dd, *J* = 3.38,2.55 Hz, 1H, ArH), 7.11–7.08 (m, 1H, ArH), 6.80 (d, *J* = 7.89 Hz, 1H, ArH), 6.72 (d, *J* = 8.18 Hz, 1H, ArH), 6.65–6.62 (m, 2H, ArH), 6.49 (d, *J* = 1.91 Hz, 1H, ArH), 6.37 (dd, *J* = 3.97, 1.88 Hz, 1H, ArH), 6.16 (s, 1H, C4-H), 3.82 (s, 3H, OMe), 3.71 (s, 3H, OMe), 2.89 (bs, 4H, 2CH_2_), 2.87 (t, *J* = 6.87 Hz, 2H, CH_2_), 2.75 (t, *J* = 7.16 Hz, 2H, CH_2_); ^13^C NMR (400 MHz, CDCl_3_): *δ* = 157.36 (d, *J*_F,C_ = 251.45 Hz, CF), 153.4, 147.5, 147.4, 144.8, 144.7, 144.3, 136.0 (d, *J*_F,C_ = 3.44 Hz, C), 133.9, 131.7, 127.9, 125.9, 120.8, 120.6, 120.5, 116.5, 116.3, 114.6, 111.8, 111.6, 105.1, 55.0, 54.8, 35.0, 34.9, 29.7, 27.5; HR-ESI(-)MS m/z 495.1489 [M–H]^−^ (calcd for [C_27_H_25_ClFN_2_O_4_]^−^, m/z 495.1492).

#### 1-(4-(Triflouromethyl)phenyl)-3,5-bis-[2-(4-hydroxy-3-methoxy)-phenylethyl]-1H-pyrazole (**14**)

Orange oil, yield: 41%. ^1^H NMR (500 MHz, CDCl_3_ *δ* = 7.73 (d, *J* = 7.74 Hz, 2H, ArH), 7.36 (d, *J* = 7.87 Hz, 2H, ArH), 6.79 (d, *J* = 1.71 Hz, 1H, ArH), 6.73 (d, *J* = 7.89 Hz, 1H, ArH), 6.65–6.61 (m, 2H, ArH), 6.47 (d, *J* = 1.74 Hz, 1H, ArH), 6.39 (dd, *J* = 1.67, 4.11 Hz, 1H, ArH), 6.18 (s, 1H, C4-H), 3.81 (s, 3H, OMe), 3.68 (s, 3H, OMe), 2.94 (t, *J* = 7.61 Hz, 2H, CH_2_), 2.90 (bs, 4H, 2CH_2_), 2.73 (t, *J* = 7.61 Hz, 2H, CH_2_); ^13^C NMR (400 MHz, CDCl_3_): *δ* = 153.8, 147.5, 147.4, 144.7, 144.6, 144.3, 142.3, 132.9, 131.7, 129.3, 129.1, 125.8 (q, *J*_F,C_ = 11.35 Hz, CF_3_), 125.6, 125.0, 122.9, 120.6, 120.5, 114.7, 111.8, 111.5, 110.7, 105.7, 55.0, 54.8, 35.0, 34.8, 29.7, 27.8; HR-ESI(-)MS m/z 511.1845 [M–H]^−^ (calcd for [C_28_H_26_F_3_N_2_O_4_]^−^, m/z 511.1850).

#### 1-(4-Triflouromethoxy)phenyl)-3,5-bis-[2-(4-hydroxy-3-methoxy)-phenylethyl]-1H-pyrazole (**15**)

Orange oil, yield: 38%. ^1^H NMR (500 MHz, CDCl_3_): *δ* = 7.35 (d, *J* = 8.37 Hz, 2H, ArH), 7.36 (d, *J* = 6.95 Hz, 2H, ArH), 6.79 (d, *J* = 1.70 Hz, 1H, ArH), 6.72 (d, *J* = 7.98 Hz, 1H, ArH), 6.65–6.62 (m, 2H, ArH), 6.50 (d, *J* = 1.73 Hz, 1H, ArH), 6.39 (dd, *J* = 4.09, 1.67 Hz, 1H, ArH), 6.16 (s, 1H, C4-H), 3.82 (s, 3H, OMe), 3.71 (s, 3H, OMe), 2.89 (bs, 6H, 3CH_2_), 2.74 (t, *J* = 7.04 Hz, 2H, CH_2_); ^13^C NMR (400 MHz, CDCl_3_): *δ* = 153.8, 148.4, 148.3, 147.5, 14.4, 144.6, 144.60, 144.3, 138.0, 133.0, 131.8, 127.9, 121.4, 121.2, 120.2, 120.2, 119.4, 114.0, 114.6, 111.8, 111.6, 105.1, 54.9, 54.8, 35.0, 34.7, 29.7, 27.7; HR-ESI(-)MS m/z 527.1802 [M–H]^−^ (calcd for [C_28_H_26_F_3_N_2_O_5_]^−^, m/z 527.1799).

#### 1-(4-Nitrophenyl)-3,5-bis-[2-(4-hydroxy-3-methoxy)-phenylethyl]-1H-pyrazole (**16**)

Red oil, yield: 40%. ^1^H NMR (500 MHz, CDCl_3_): *δ* = 8.27–8.24 (m, 2H, ArH), 7.47–7.43 (m, 2H, ArH), 6.80 (s, 1H, ArH), 6.72 (d, *J* = 8.11 Hz, 1H, ArH), 6.65–6.64 (m, 1H, ArH), 6.62–6.60 (m, 1H, ArH), 6.51–6.50 (m, 1H, ArH), 6.40–6.38 (m, 1H, ArH), 6.21 (d, *J* = 1.72 Hz, 1H, C4-H), 3.82 (d, *J* = 1.57 Hz, 3H, OMe), 3.68 (d, *J* = 1.57 Hz, 3H, OMe), 3.03–3.00 (m, 2H, CH_2_), 2.90 (bs, 4H, 2CH_2_), 2.78–2.75 (m, 2H, CH_2_); ^13^C NMR (400 MHz, CDCl_3_): *δ* = 154.4, 147.5, 147.4, 146.2, 144.9, 144.8, 144.7, 144.5, 144.4, 132.8, 131.5, 125.1, 124.1, 120.6, 120.5, 114.7, 111.8, 111.5, 106.5, 55.0, 54.8, 34.9, 34.8, 29.8, 28.0; HR-ESI(-)MS m/z 488.1825 [M–H]^−^ (calcd for [C_27_H_26_N_3_O_6_]^−^, m/z 488.1827).

#### 1-(3-Nitrophenyl)-3,5-bis-[2-(4-hydroxy-3-methoxy)-phenylethyl]-1H-pyrazole (**17**)

Yellow oil, yield: 26%. ^1^H NMR (500 MHz, CDCl_3_): *δ* = 8.21–8.19 (m, 1H, ArH), 8.00 (t, *J* = 1.90 Hz, 2H, ArH), 7.63 (t, *J* = 8.01 Hz, 1H, ArH), 7.56–7.54 (m, 1H, ArH), 6.80 (d, *J* = 1.80 Hz, 1H, H1), 6.72 (d, *J* = 7.98 Hz, 1H, H1), 6.66–6.64 (m, 1H, ArH), 6.57 (d, *J* = 7.83 Hz, 1H, H1), 6.46 (d, *J* = 1.94 Hz, 1H, H1), 6.33 (dd, *J* = 4.01, 1.81 Hz, 1H, C4-H), 3.82 (s, 3H, OMe), 3.67 (3H, s, OMe), 2.97 (t, *J* = 7.19 Hz, 2H, CH_2_), 2.91 (bs, 4H, 2CH_2_), 2.74 (t, *J* = 7.28 Hz, 2H, CH_2_); ^13^C NMR (400 MHz, CDCl_3_): *δ* = 154.1, 148.3, 147.4, 147.3, 144.8, 144.6, 144.3, 140.1, 132.9, 131.5, 130.8, 129.8, 121.8, 120.6, 120.5, 119.8, 114.7, 114.6, 111.8, 111.4, 105.9, 54.9, 54.7, 35.0, 34.9, 29.7, 27.7; HR-ESI(-)MS m/z 488.1824 [M–H]^−^ (calcd for [C_27_H_26_N_3_O_6_]^−^, m/z 488.1827).

#### 1-(Benzothiazol-2-yl)-3,5-bis-[2-(4-hydroxy-3-methoxy)-phenylethyl]-1H-pyrazole (**18**)

Gray solid, yield: 36%. ^1^H NMR (500 MHz, CDCl_3_): *δ* = 7.89 (d, *J* = 8.02 Hz, 1H, ArH), 7.85 (d, *J* = 7.96 Hz, 1H, ArH), 7.49–7.46 (m, 1H, ArH), 7.38–7.34 (m, 1H, ArH), 6.90–6.87 (m, 2H, ArH), 6.83–6.76 (m, 4H, ArH), 6.04 (s, 1H, C4-H), 3.91 (s, 3H, OMe), 3.90 (s, 3H, OMe), 3.55 (t, *J* = 7.69 Hz, 2H, CH_2_), 3.01 (t, *J* = 7.68 Hz, 2H, CH_2_), 2.97 (bs, 4H, 2CH_2_); ^13^C NMR (400 MHz, CDCl_3_): *δ* = 161.2, 155.5, 151.6, 146.4, 146.2, 143.9, 143.8, 133.3, 133.1, 133.0, 130.9, 128.8, 126.1, 124.5, 122.3, 121.2, 121.1, 121.0, 114.3, 114.2, 111.0, 108.8, 55.9, 55.9, 34.7, 34.5, 30.3, 29.9; HR-ESI(-)MS m/z 500.1649 [M–H]^−^ (calcd for [C_28_H_26_N_3_O_4_S]^−^, m/z 500.1649).

### Cytotoxic Activity Assay

Human lung adenocarcinoma (A549), human cervical carcinoma (HeLa) and human breast carcinoma (MCF-7) cell lines were obtained from Kunming Institute of Zoology, Chinese Academy of Sciences (Kunming, China). The cells were cultured in RPMI-1640 medium supplemented with 10% fetal bovine serum in a humidified atmosphere with 5% CO_2_ at 37 °C throughout the assay. Cell viability was estimated by the MTT colorimetric assay [29]. The test compounds in DMSO (10 mg/mL) were serially diluted with culture medium. Test cells (100 μL, 5 × 104 cells/mL) were seeded into a 96-well microplate and incubated about 24 h for cell implantation; then the supernatant was removed, and 100 μL of fresh medium and 100 μL of medium containing a test compound or vehicle control (DMSO) were added. The blank control contained 200 μL of medium without cells. The final concentrations of each compound in the wells were 50, 10, 2, 0.4, 0.08, and 0.016 μg/mL, and the experiments for each concentration were performed in quadruplicate. THC was used as a positive control. Cells were further incubated for 72 h and then treated with MTT (20 μL, 5 mg/mL in DMSO) and shaken for 15 min. After another 4 h of incubation, the supernatant per well was removed and 150 μL of DMSO was added to dissolve the blue formazan crystals. The optical density (OD) of each well was measured on a Genois microplate reader at a wavelength of 570 nm. The inhibitory rate of cell growth was calculated according to the following formula: Inhibition rate (%) = {1 − (OD_treated_ − OD_control_)/(OD_control_ − OD_blank_)} × 100%. IC_50_ values were determined by nonlinear regression analysis of logistic dose–response curves (SPSS 16.0 statistic software).

## Conclusions

In conclusion, we have synthesized a series of pyrazole derivatives of tetrahydrocurcumin (THC). The prepared compounds have been evaluated for their cell proliferation-inhibitory activity against three cancer cell lines including A549, HeLa and MCF-7. Most derivatives are significantly more active than the parent compound THC. A few aryl-substituted derivatives show IC_50_ values below 10 µM against all tested cell lines. The most active compound (**8**) with an *N*-4-bromo-phenyl substituent has IC_50_ values ranging from 5.8 to 9.8 μM. Further research is needed in order to understand their anticancer mechanism and study more thoroughly the structure–activity relationship of these pyrazole derivatives.

## Abbreviations

A549: Human lung adenocarcinoma
Cisplatin: *cis*-Diamminedichloridoplatinum (II)
DCM: Dichloromethane
DMSO: Dimethyl sulfoxide
EtOAc: Ethyl acetate
HeLa: Human cervical carcinoma
MCF-7: Human breast carcinoma
MDR: Multiple drug resistance
MS: Mass spectroscopy
MTT: 3-(4,5-Dimethylthiazol-2-yl)-2,5-diphenyltetrazolium bromide
NMR: Nuclear magnetic resonance
PtO_2_: Platinum (IV) oxide
THC: Tetrahydrocurcumin
TLC: Thin-layer chromatography

## References

[1] Rao B, Prasad E, Deepthi SS, Ansari IA (2014). Arch. Pharm. Chem. Life Sci..

[2] Shehzad A, Lee J, Lee YS (2013). BioFactors.

[3] Panda K, Chakraborty D, Sarkar I, Khan T, Sa G (2017). J. Exp. Pharmacol..

[4] Labbozzetta M, Notarbartolo M, Poma P, Maurici A, Inguglia L, Marchetti P, Rizzi M, Baruchello R, Simoni D, D’Alessandro N (2009). Ann. N. Y. Acad. Sci..

[5] Xiao H, Xiao Q, Zhang K, Zuo X, Shrestha UK (2010). Ann. Hematol..

[6] Kang N, Wang MM, Wang YH, Zhang ZN, Cao HR, Lv YH, Yang Y, Fan PH, Qiu F, Cao XM (2014). Food Chem. Toxicol..

[7] Mohri K, Watanable Y, Yoshida Y, Satoh M, Isobe K, Sugimoto N, Tsudac Y (2003). Chem. Pharm. Bull..

[8] Sugiyamu Y, Kawakishi S, Osawa T (1996). Biochem. Pharmacol..

[9] Yodkeeree S, Garbisa S, Limtrakul P (2008). Acta Pharmacol. Sin..

[10] Yoysungnoen B, Bhattarakosol P, Patumraj S, Changtam C (2016). Biomed. Res. Int..

[11] Limtrakul P, Chearwae W, Shukla S, Phisalphong C, Ambudkar SV (2007). Mol. Cell. Biochem..

[12] Song K, Park JY, Lee S, Lee D, Jang HJ, Kim SN, Ko H, Kim HY, Lee JW, Hwang GS, Kang KS, Yamabe N (2015). Planta Med..

[13] Majeed M, Badmaev V, Shivakumar U, Rajendran R (1995). Curcuminoids: Antioxidant Phytonutrients.

[14] Muthumani M, Miltonprabu S (2015). Chem. Biol. Interact..

[15] Vyas A, Dandawate P, Padhye S, Ahmad A, Sarkar F (2013). Curr. Pharm. Des..

[16] Marchiani A, Rozzo C, Fadda A, Delogu G, Ruzza P (2014). Curr. Med. Chem..

[17] Huang MT, Newmark L, Frenkel KJ (1997). Cell Biochem. Suppl..

[18] Rao AB, Prasad E, Deepthi SS, Ansari IA (2014). Arch. Pharm. Chem. Life Sci..

[19] Plyduang T, Lomlim L, Yuenyongsawad S, Wiwattanapatapee R (2014). Eur. J. Pharm. Biopharm..

[20] Manjunatha JR, Bettadaiah BK, Negi PS, Srinivas P (2013). Food Chem..

[21] Manjunatha JR, Bettadaiah BK, Negi PS, Srinivas P (2013). Food Chem..

[22] Nelson KM, Dahlin JL, Bisson J, Graham J, Pauli GF, Walters MA (2017). J. Med. Chem..

[23] Mohareb RM, Omran FA (2012). Steroids.

[24] Zheng LW, Li Y, Ge D, Zhao B-X, Liu YR, Lv HS, Ding J, Miao JY (2010). Bioorg. Med. Chem. Lett..

[25] Tong Y, Claiborne A, Pyzytulinska M, Tao ZF, Stewart KD, Kovar P, Chen Z, Credo RB, Guan R, Merta PJ, Zhang H, Bouska J, Everitt EA, Murry BP, Hickman D, Stratton TJ, Wu J, Rosenberg SH, Sham HL, Sowin TJ (2007). Bioorg. Med. Chem. Lett..

[26] Fustero S, Anchez-Roselló M, Barrio P, Simón-Fuentes A (2011). Chem. Rev..

[27] Flynn DL, Belliotti TR, Boctor AM, Connor DT, Kostlan CR, Nies DE, Ortwine DF, Schrier DJ, Sircar JC (1991). J. Med. Chem..

[28] Mosman T, Immun J (1983). Methods.

[29] Shi JF, Wu P, Jiang ZH, Wei XY (2014). Eur. J. Med. Chem..

